# Navegando la transición: desafíos y oportunidades en la atención de los pacientes con errores innatos de la inmunidad

**DOI:** 10.7705/biomedica.7815

**Published:** 2024-12-23

**Authors:** Andrés Felipe Zea-Vera, Lina María Castaño-Jaramillo

**Affiliations:** 1 Departamento de Microbiología, Facultad de Salud, Universidad del Valle, Cali, Colombia; Genetic Immunotherapy Section, Laboratory of Clinical Immunology and Microbiology, National Institute of Allergy and Infectious Diseases, National Institutes of Health, Bethesda, MD, USA Departamento de Microbiología, Facultad de Salud Universidad del Valle Cali Colombia; 2 Servicio de Inmunología Clínica y Alergia Pediátrica, Fundación Hospital Pediátrico de La Misericordia, Bogotá, D. C., Colombia Servicio de Inmunología Clínica y Alergia Pediátrica Fundación Hospital Pediátrico de La Misericordia Bogotá, D. C. Colombia

La transición a la vida adulta es un hito que representa un sueño para unos y un temor para otros. Para los pacientes con errores innatos de la inmunidad y otras enfermedades crónicas, este proceso implica desafíos adicionales al cambiar su cuidado de las especialidades pediátricas al de los servicios de adultos.

Uno de los principales problemas es la escasez de médicos especializados en inmunología de adultos. En Colombia, muchos de los especialistas en inmunología comienzan su carrera en pediatría, lo que resulta en una proporción limitada de médicos con experiencia en adultos. Esto crea un vacío en la atención especializada para estos pacientes a medida que avanzan hacia la adultez.

Con la adolescencia y la vida adulta vienen nuevos retos médicos: comorbilidades asociadas con la enfermedad subyacente, complicaciones derivadas del uso prolongado de medicamentos, factores de riesgo como el tabaquismo y el consumo de alcohol, y el inicio de la vida sexual. Estos elementos complejizan la gestión de su salud en la adultez.

La transición del cuidado pediátrico al de adultos no debe verse como un final, sino como una oportunidad y una historia de éxito al lograr que estos pacientes, que antes frecuentemente fallecían en la edad pediátrica, alcancen la vida adulta. Cada vez más niños con errores innatos de la inmunidad llegan a la adultez, y es imperativo que los médicos de adultos se familiaricen con estos errores innatos, dado que su prevalencia en adultos está aumentando y las complicaciones asociadas se hacen más evidentes. Un desafío crucial es garantizar la continuidad de la atención en salud. Los pacientes frecuentemente enfrentan demoras significativas para acceder a los servicios de adultos debido a dificultades administrativas, lo que puede resultar en interrupciones en su tratamiento. Este retraso en la transición puede tener un impacto negativo en su salud y bienestar general.

La transición a la atención de adultos también es emocionalmente desafiante para los pacientes y sus familias, y también, para los médicos tratantes. Despedirse de los equipos médicos que han estado involucrados en su cuidado desde el diagnóstico puede ser un proceso difícil. Es esencial fomentar la autonomía del paciente desde la adolescencia, promoviendo su participación en la gestión de su propio cuidado, incluyendo la administración de medicamentos, el agendamiento de citas médicas, y la respuesta activa durante las consultas; en algunos servicios de adultos pueden tener políticas restrictivas sobre los acompañantes.

Otro aspecto por considerar es la empleabilidad de estos pacientes. La mayor demanda de servicios de salud y las frecuentes incapacidades debido a citas médicas u hospitalizaciones, pueden dificultar su competencia en el mercado laboral. Los pacientes con errores innatos de la inmunidad enfrentan un sinnúmero de barreras psicosociales, que incluyen el estigma asociado con sus enfermedades, la incertidumbre sobre su futuro y la dependencia económica prolongada de sus familias. Como inmunólogos, nuestro rol se extiende a proporcionar orientación sobre la planificación familiar, el uso de métodos anticonceptivos y el manejo de la fertilidad, en aquellos con tratamientos inmunosupresores o con riesgo de transmitir la enfermedad a sus descendientes.

La transición del cuidado pediátrico al de adultos representa, no solo un cambio de entorno médico, sino un momento crítico para reevaluar las necesidades a largo plazo de estos pacientes, por lo cual se promueve un enfoque holístico que abarque todos los aspectos de su bienestar. A diferencia de lo que ocurre en pediatría, el manejo de los adultos se caracteriza por un mayor enfoque en la prevención de complicaciones a largo plazo y por la gestión integral de las comorbilidades. Las infecciones recurrentes y las secuelas inflamatorias -muchas de las cuales se desarrollan de manera insidiosa durante la infancia- pueden emerger como problemas predominantes en la adultez y deben abordarse de manera proactiva para evitar un deterioro significativo de la calidad de vida de los pacientes.La transición ofrece una oportunidad para replantear el uso de terapias inmunomoduladoras o de reemplazo en el contexto de nuevos factores de riesgo, como el envejecimiento prematuro del sistema inmunitario o la mayor propensión a las enfermedades autoinmunitarias y a las neoplasias. Para los inmunólogos clínicos de adultos, esto implica, no solo mantener el control de la enfermedad subyacente, sino también, implementar estrategias de tamizaje y seguimiento para detectar precozmente estas complicaciones y adaptar el esquema terapéutico a la nueva realidad del paciente.

En este proceso, resulta fundamental un modelo de cuidado multidisciplinario que involucre especialistas en reumatología, neumología, nefrología y otros, según las manifestaciones clínicas en cada caso. Los inmunólogos de adultos deben estar preparados para liderar este equipo, coordinando y comunicándose de manera efectiva, para garantizar que todas las necesidades médicas sean satisfechas sin generar duplicidad de servicios ni fragmentación del cuidado.

El abordaje de la salud mental es otro pilar de la transición. Es vital implementar estrategias de apoyo emocional y de manejo del estrés, pues estos pacientes han crecido enfrentando enfermedades crónicas que han impactado su autoestima y autonomía. El desarrollo de habilidades de afrontamiento y la integración de la salud mental en el seguimiento regular, son componentes esenciales para favorecer la adhesión al tratamiento y mejorar su calidad de vida.

A medida que el campo de la inmunología avanza en Colombia, es crucial, no solo mejorar las técnicas de diagnóstico y la disponibilidad de nuevos tratamientos, sino también, desarrollar estrategias de transición bien estructuradas y coordinadas. Esto incluye establecer programas de transición específicos, fortalecer la comunicación entre los equipos pediátricos y los de adultos, y proporcionar apoyo psicosocial adecuado. El involucrar a los pacientes en la planificación de su transición con anticipación, y ofrecer recursos y apoyo continuo, son pasos vitales para garantizar que esta etapa se maneje con la atención y la consideración necesarias.

Por último, es crucial que las sociedades científicas y las instituciones académicas promuevan la formación de inmunólogos para adultos en Colombia, fomentando la creación de programas académicos y de certificación que permitan llenar el actual vacío de conocimiento. La inclusión de módulos específicos sobre los errores innatos de la inmunidad en las especialidades de medicina interna e inmunología clínica es imperativa, para garantizar que los médicos en formación comprendan las particularidades de estas patologías y que estén preparados para atender una población que continuará en expansión.

La transición -más que un simple cambio de consultorio- debe ser vista como una fase en la que podemos acompañar a los pacientes a alcanzar su máximo potencial en la vida adulta. Nuestra misión, como inmunólogos clínicos, no se limita a ofrecer el tratamiento médico, sino a construir puentes que permitan a estos pacientes avanzar hacia un futuro más saludable y lleno de posibilidades.
